# 2-(4-Chloro­benzo­yl)-3,6-dimethoxy­naphthalene

**DOI:** 10.1107/S1600536808004704

**Published:** 2008-02-22

**Authors:** Kosuke Nakaema, Akiko Okamoto, Masahiro Imaizumi, Keiichi Noguchi, Noriyuki Yonezawa

**Affiliations:** a Department of Organic and Polymer Materials Chemistry, Tokyo University of Agriculture & Technology, Koganei, Tokyo 184-8588, Japan; b Section Manager, Group I, Section III, Functional Chemicals Research Laboratory, Nippon Kayaku Co. Ltd, Shimo 3-chome, Kita-ku, Tokyo 115-0042, Japan; c Instrumentation Analysis Center, Tokyo University of Agriculture & Technology, Koganei, Tokyo 184-8588, Japan

## Abstract

In the title compound, C_19_H_15_ClO_3_, the inter­planar angle between the naphthalene and benzene ring systems is 62.67 (6)°. The carbonyl group is twisted from both ring planes, with torsion angles of −44.9 (2)° with respect to the naphthalene ring and −26.7 (2)° with respect to the phenyl­ene ring. There is an inter­molecular hydrogen bond between an H atom of one meth­oxy group and the O atom of the second meth­oxy group, forming chains along the *ac* diagonal.

## Related literature

For related literature, see: Ahn *et al.* (2003 ▶); Allen *et al.* (1998 ▶); Chen *et al.* (2005 ▶); Crasto & Stevens (1998 ▶, 2002 ▶); Lorenzetti *et al.* (2005 ▶); Nakaema *et al.* (2007 ▶); Su *et al.* (2004 ▶); Wang & Guen (1995 ▶).
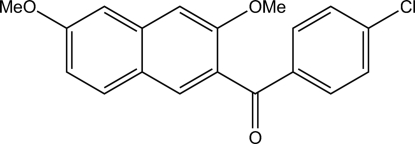


## Experimental

### 

#### Crystal data

C_19_H_15_ClO_3_
*M* *_r_* = 326.76
Monoclinic, 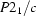

*a* = 8.1894 (5) Å
*b* = 20.5251 (13) Å
*c* = 9.9098 (7) Å
β = 106.358 (4)°
*V* = 1598.29 (18) Å^3^
*Z* = 4
Cu *K*α radiation
μ = 2.22 mm^−1^
*T* = 296 K
0.50 × 0.25 × 0.10 mm

#### Data collection

Rigaku R-AXIS RAPID diffractometer
Absorption correction: numerical (*NUMABS*; Higashi, 1999 ▶) *T*_min_ = 0.458, *T*_max_ = 0.801
30087 measured reflections
2917 independent reflections
2652 reflections with *I* > 2σ(*I*)
*R*_int_ = 0.049

#### Refinement

*R*[*F* ^2^ > 2σ(*F* ^2^)] = 0.033
*wR*(*F* ^2^) = 0.100
*S* = 1.07
2917 reflections
211 parameters
H-atom parameters constrained
Δρ_max_ = 0.17 e Å^−3^
Δρ_min_ = −0.28 e Å^−3^

### 

Data collection: *PROCESS-AUTO* (Rigaku, 1998 ▶); cell refinement: *PROCESS-AUTO*; data reduction: *CrystalStructure* (Rigaku/MSC, 2004 ▶); program(s) used to solve structure: *SIR92* (Altomare *et al.*, 1994 ▶); program(s) used to refine structure: *SHELXL97* (Sheldrick, 2008 ▶); molecular graphics: *ORTEPIII* (Burnett & Johnson, 1996 ▶); software used to prepare material for publication: *SHELXL97*.

## Supplementary Material

Crystal structure: contains datablocks I, global. DOI: 10.1107/S1600536808004704/fl2189sup1.cif

Structure factors: contains datablocks I. DOI: 10.1107/S1600536808004704/fl2189Isup2.hkl

Additional supplementary materials:  crystallographic information; 3D view; checkCIF report

## Figures and Tables

**Table 1:** Hydrogen-bond geometry (Å, °)

*D*—H⋯*A*	*D*—H	H⋯*A*	*D*⋯*A*	*D*—H⋯*A*
C18—H18*C*⋯O3^i^	0.96	2.51	3.460 (2)	171

Symmetry code: (i) 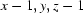.

**Fig. 1.**
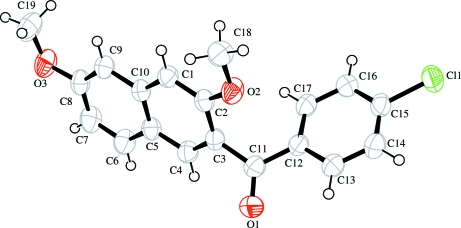
Molecular structure of (I), with the atom-labeling scheme and displacement ellipsoids drawn at the 50% probability level.

**Fig. 2.**
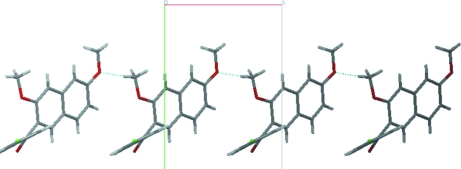
A partial packing diagram of the title compound, viewed down the c axis. The dashed lines indicate hydrogen bonds.

## supplementary crystallographic information

### Comment

Naphthalene derivatives, such as 1,5-disubstituted and 2,6-disubstituted naphthalenes, have been used widely as key building blocks of functional organic compounds such as liquid crystals and electric materials (Su *et al.*, 2004; Ahn *et al.*, 2003; Lorenzetti *et al.*, 2005; Chen *et al.*, 2005). Recently, 1,8-disubstituted naphthalenes have received much attention as unique structured aromatic core compounds, exemplified by dendron cores and supramolecular building blocks (Wang & Guen, 1995; Allen *et al.*, 1998; Crasto & Stevens, 1998, 2002).

In this paper, the structural characteristics of the title compound, which is one of the products of electrophilic aromatic aroylation of 2,7-dimethoxynaphthalene, is reported and discussed. The authors have recently reported the crystal structure of the 1,8-diaroylated derivative of 2,7-dimethoxynaphthalene as the product of regioselective electrophilic aromatic aroylation of 2,7-dimethoxynaphthalene with 4-chlorobenzoic acid (Nakaema *et al.*, 2007). As 3-substituted naphthalene compounds are generally regarded to be thermodynamically more stable than the corresponding 1-positioned isomeric molecules, the title molecule is of interest from both the stereochemical features of its conformation and the thermodynamic aspects of its molecular structure.

An *ORTEPIII* (Burnett & Johnson, 1996) plot of the title molecule, (I), is displayed in Fig. 1. The 4-chlorobenzoyl group is twisted away from the attached naphthalene ring. The interplanar angle between the best planes of the chlorophenyl ring and the naphthalene ring is 62.67 (6)°. The torsion angle between the carbonyl group and the naphthalene ring is relatively large [C4—C3—C11—O1 = -44.9 (2)°] and that between 4-chlorophenyl group and carbonyl group is rather small [O1—C11—C12—C13 = -26.7 (2)°].

The crystal packing is stabilized mainly by van der Waals interactions. In addition, there is a C—H···O hydrogen bond between a hydrogen of the 2-methoxy group which is situated adjacent to the chlorobenzoyl group, and the ethereal oxygen of the 7-methoxy group in a neighboring molecule that could also contribute the stabilization of the crystal packing (Table 1, Figure 2).

### Experimental

The title compound was prepared by electrophilic aromatic aroylation reaction of 2,7-dimethoxynaphthalene with 4-chlorobenzoic acid. Yellow single crystals suitable for X-ray diffraction were obtained by recrystallization from ethanol and ethyl acetate.

### Refinement

All the H atoms were found in difference maps and were subsequently refined as riding atoms, with C—H = 0.93 (aromatic) and 0.96 (methyl) Å, and *U*_iso_(H) = 1.2*U*_eq_(C).

### Crystal data

**Table:**

C_19_H_15_ClO_3_	*F*_000_ = 680
*M**_r_* = 326.76	*D*_x_ = 1.358 Mg m^−^^3^
Monoclinic, *P*2_1_/*c*	Melting point = 424.8–425.2 K
Hall symbol: -P 2ybc	Cu *K*α radiation λ = 1.54187 Å
*a* = 8.1894 (5) Å	Cell parameters from 28831 reflections
*b* = 20.5251 (13) Å	θ = 4.3–68.2º
*c* = 9.9098 (7) Å	µ = 2.22 mm^−^^1^
β = 106.358 (4)º	*T* = 296 K
*V* = 1598.29 (18) Å^3^	Platelet, colorless
*Z* = 4	0.50 × 0.25 × 0.10 mm

### Data collection

**Table:**

Rigaku R-AXIS RAPID diffractometer	2917 independent reflections
Radiation source: rotating anode	2652 reflections with *I* > 2σ(*I*)
Monochromator: graphite	*R*_int_ = 0.049
Detector resolution: 10.00 pixels mm^-1^	θ_max_ = 68.2º
*T* = 296 K	θ_min_ = 4.3º
ω scans	*h* = −9→9
Absorption correction: numerical(NUMABS; Higashi, 1999)	*k* = −24→24
*T*_min_ = 0.458, *T*_max_ = 0.801	*l* = −11→11
30087 measured reflections	

### Refinement

**Table:**

Refinement on *F*^2^	Hydrogen site location: difference Fourier map
Least-squares matrix: full	H-atom parameters constrained
*R*[*F*^2^ > 2σ(*F*^2^)] = 0.033	*w* = 1/[σ^2^(*F*_o_^2^) + (0.054*P*)^2^ + 0.2906*P*] where *P* = (*F*_o_^2^ + 2*F*_c_^2^)/3
*wR*(*F*^2^) = 0.100	(Δ/σ)_max_ < 0.001
*S* = 1.07	Δρ_max_ = 0.17 e Å^−^^3^
2917 reflections	Δρ_min_ = −0.27 e Å^−^^3^
211 parameters	Extinction correction: SHELXL97 (Sheldrick, 1997), Fc^*^=kFc[1+0.001xFc^2^λ^3^/sin(2θ)]^-1/4^
Primary atom site location: structure-invariant direct methods	Extinction coefficient: 0.0039 (4)
Secondary atom site location: difference Fourier map	

### Special details

**Table:**

Geometry. All e.s.d.'s (except the e.s.d. in the dihedral angle between two l.s. planes) are estimated using the full covariance matrix. The cell e.s.d.'s are taken into account individually in the estimation of e.s.d.'s in distances, angles and torsion angles; correlations between e.s.d.'s in cell parameters are only used when they are defined by crystal symmetry. An approximate (isotropic) treatment of cell e.s.d.'s is used for estimating e.s.d.'s involving l.s. planes.
Refinement. Refinement of *F*^2^ against ALL reflections. The weighted *R*-factor *wR* and goodness of fit *S* are based on *F*^2^, conventional *R*-factors *R* are based on *F*, with *F* set to zero for negative *F*^2^. The threshold expression of *F*^2^ > σ(*F*^2^) is used only for calculating *R*-factors(gt) *etc*. and is not relevant to the choice of reflections for refinement. *R*-factors based on *F*^2^ are statistically about twice as large as those based on *F*, and *R*- factors based on ALL data will be even larger.

### Fractional atomic coordinates and isotropic or equivalent isotropic displacement parameters (Å^2^)

**Table:**

	*x*	*y*	*z*	*U*_iso_*/*U*_eq_	
Cl1	−0.25848 (6)	0.44273 (2)	−0.48382 (4)	0.06829 (17)	
O1	−0.25367 (15)	0.47599 (6)	0.18888 (12)	0.0662 (3)	
O2	−0.21482 (13)	0.30244 (5)	0.06521 (11)	0.0560 (3)	
O3	0.44684 (14)	0.21975 (6)	0.70829 (12)	0.0662 (3)	
C1	0.01126 (17)	0.27069 (7)	0.26978 (14)	0.0452 (3)	
H1	0.0024	0.2272	0.2421	0.054*	
C2	−0.09091 (17)	0.31627 (7)	0.18563 (14)	0.0439 (3)	
C3	−0.07602 (17)	0.38337 (7)	0.22433 (14)	0.0441 (3)	
C4	0.03617 (18)	0.40089 (7)	0.35010 (15)	0.0478 (3)	
H4	0.0430	0.4444	0.3770	0.057*	
C5	0.14146 (17)	0.35510 (7)	0.44000 (14)	0.0457 (3)	
C6	0.2559 (2)	0.37226 (8)	0.57153 (17)	0.0573 (4)	
H6	0.2642	0.4155	0.6006	0.069*	
C7	0.3536 (2)	0.32631 (8)	0.65574 (17)	0.0602 (4)	
H7	0.4280	0.3384	0.7416	0.072*	
C8	0.34269 (17)	0.26071 (8)	0.61388 (15)	0.0515 (4)	
C9	0.23401 (16)	0.24175 (7)	0.48760 (15)	0.0475 (3)	
H9	0.2284	0.1982	0.4606	0.057*	
C10	0.13031 (16)	0.28874 (7)	0.39836 (14)	0.0427 (3)	
C11	−0.18305 (18)	0.43524 (7)	0.13539 (15)	0.0474 (3)	
C12	−0.19823 (17)	0.43754 (6)	−0.01795 (15)	0.0446 (3)	
C13	−0.34026 (19)	0.46603 (8)	−0.11003 (16)	0.0559 (4)	
H13	−0.4242	0.4839	−0.0747	0.067*	
C14	−0.3593 (2)	0.46831 (8)	−0.25247 (17)	0.0579 (4)	
H14	−0.4556	0.4870	−0.3132	0.070*	
C15	−0.2335 (2)	0.44241 (6)	−0.30339 (15)	0.0498 (3)	
C16	−0.0880 (2)	0.41601 (8)	−0.21476 (16)	0.0561 (4)	
H16	−0.0017	0.4004	−0.2502	0.067*	
C17	−0.07217 (19)	0.41313 (7)	−0.07204 (16)	0.0527 (4)	
H17	0.0246	0.3945	−0.0116	0.063*	
C18	−0.2507 (2)	0.23549 (8)	0.03027 (17)	0.0576 (4)	
H18A	−0.2803	0.2140	0.1061	0.086*	
H18B	−0.1519	0.2151	0.0149	0.086*	
H18C	−0.3439	0.2324	−0.0537	0.086*	
C19	0.4318 (2)	0.15190 (9)	0.6806 (2)	0.0759 (5)	
H19A	0.4573	0.1429	0.5936	0.114*	
H19B	0.3178	0.1381	0.6738	0.114*	
H19C	0.5102	0.1288	0.7556	0.114*	

### Atomic displacement parameters (Å^2^)

**Table:**

	*U*^11^	*U*^22^	*U*^33^	*U*^12^	*U*^13^	*U*^23^
Cl1	0.1005 (4)	0.0595 (3)	0.0412 (2)	0.0008 (2)	0.0139 (2)	0.00090 (15)
O1	0.0758 (7)	0.0705 (7)	0.0523 (6)	0.0258 (6)	0.0178 (5)	0.0004 (5)
O2	0.0605 (6)	0.0511 (6)	0.0437 (5)	−0.0009 (5)	−0.0059 (5)	0.0007 (4)
O3	0.0597 (6)	0.0702 (7)	0.0541 (7)	0.0035 (5)	−0.0078 (5)	0.0142 (5)
C1	0.0489 (7)	0.0428 (7)	0.0405 (7)	−0.0011 (5)	0.0070 (6)	−0.0004 (5)
C2	0.0440 (7)	0.0491 (7)	0.0355 (7)	−0.0019 (5)	0.0063 (5)	0.0004 (5)
C3	0.0458 (7)	0.0467 (7)	0.0385 (7)	0.0014 (5)	0.0099 (6)	0.0039 (5)
C4	0.0523 (8)	0.0437 (7)	0.0454 (8)	−0.0030 (6)	0.0105 (6)	0.0002 (6)
C5	0.0450 (7)	0.0489 (7)	0.0407 (7)	−0.0052 (6)	0.0079 (6)	0.0018 (6)
C6	0.0609 (9)	0.0543 (8)	0.0480 (8)	−0.0101 (7)	0.0014 (7)	−0.0023 (7)
C7	0.0586 (9)	0.0664 (10)	0.0442 (8)	−0.0109 (7)	−0.0039 (7)	0.0006 (7)
C8	0.0436 (7)	0.0621 (9)	0.0439 (8)	−0.0021 (6)	0.0044 (6)	0.0109 (6)
C9	0.0458 (7)	0.0495 (8)	0.0442 (8)	−0.0006 (6)	0.0077 (6)	0.0047 (6)
C10	0.0401 (6)	0.0487 (7)	0.0381 (7)	−0.0029 (5)	0.0090 (5)	0.0035 (5)
C11	0.0468 (7)	0.0472 (7)	0.0467 (8)	0.0045 (6)	0.0106 (6)	0.0016 (6)
C12	0.0474 (7)	0.0405 (7)	0.0443 (8)	0.0040 (5)	0.0101 (6)	0.0039 (5)
C13	0.0526 (8)	0.0628 (9)	0.0504 (9)	0.0167 (7)	0.0117 (7)	0.0064 (7)
C14	0.0561 (8)	0.0608 (9)	0.0497 (9)	0.0109 (7)	0.0033 (7)	0.0088 (7)
C15	0.0652 (9)	0.0395 (7)	0.0417 (8)	−0.0021 (6)	0.0102 (6)	0.0031 (5)
C16	0.0638 (9)	0.0562 (8)	0.0516 (9)	0.0124 (7)	0.0218 (7)	0.0063 (7)
C17	0.0501 (7)	0.0569 (8)	0.0491 (8)	0.0138 (6)	0.0109 (6)	0.0100 (7)
C18	0.0579 (9)	0.0551 (9)	0.0509 (9)	−0.0030 (7)	0.0007 (7)	−0.0085 (7)
C19	0.0724 (11)	0.0675 (11)	0.0721 (12)	0.0099 (9)	−0.0050 (9)	0.0169 (9)

### Geometric parameters (Å, °)

**Table:**

Cl1—C15	1.7415 (15)	C8—C9	1.372 (2)
O1—C11	1.2196 (18)	C9—C10	1.4180 (19)
O2—C2	1.3610 (16)	C9—H9	0.9300
O2—C18	1.4273 (18)	C11—C12	1.490 (2)
O3—C8	1.3638 (17)	C12—C17	1.384 (2)
O3—C19	1.418 (2)	C12—C13	1.3891 (19)
C1—C2	1.3703 (19)	C13—C14	1.377 (2)
C1—C10	1.4184 (18)	C13—H13	0.9300
C1—H1	0.9300	C14—C15	1.376 (2)
C2—C3	1.425 (2)	C14—H14	0.9300
C3—C4	1.3718 (19)	C15—C16	1.377 (2)
C3—C11	1.4980 (18)	C16—C17	1.385 (2)
C4—C5	1.4098 (19)	C16—H16	0.9300
C4—H4	0.9300	C17—H17	0.9300
C5—C10	1.419 (2)	C18—H18A	0.9600
C5—C6	1.419 (2)	C18—H18B	0.9600
C6—C7	1.360 (2)	C18—H18C	0.9600
C6—H6	0.9300	C19—H19A	0.9600
C7—C8	1.404 (2)	C19—H19B	0.9600
C7—H7	0.9300	C19—H19C	0.9600
			
C2—O2—C18	117.72 (11)	O1—C11—C3	120.14 (13)
C8—O3—C19	117.97 (13)	C12—C11—C3	119.31 (12)
C2—C1—C10	121.05 (13)	C17—C12—C13	118.41 (13)
C2—C1—H1	119.5	C17—C12—C11	121.76 (12)
C10—C1—H1	119.5	C13—C12—C11	119.81 (13)
O2—C2—C1	124.60 (12)	C14—C13—C12	121.32 (14)
O2—C2—C3	115.07 (11)	C14—C13—H13	119.3
C1—C2—C3	120.30 (12)	C12—C13—H13	119.3
C4—C3—C2	118.85 (12)	C15—C14—C13	118.88 (14)
C4—C3—C11	118.65 (13)	C15—C14—H14	120.6
C2—C3—C11	122.47 (12)	C13—C14—H14	120.6
C3—C4—C5	122.22 (13)	C14—C15—C16	121.41 (14)
C3—C4—H4	118.9	C14—C15—Cl1	119.44 (12)
C5—C4—H4	118.9	C16—C15—Cl1	119.15 (12)
C4—C5—C10	118.58 (12)	C15—C16—C17	118.92 (14)
C4—C5—C6	122.91 (13)	C15—C16—H16	120.5
C10—C5—C6	118.50 (13)	C17—C16—H16	120.5
C7—C6—C5	120.92 (15)	C12—C17—C16	120.98 (13)
C7—C6—H6	119.5	C12—C17—H17	119.5
C5—C6—H6	119.5	C16—C17—H17	119.5
C6—C7—C8	120.41 (14)	O2—C18—H18A	109.5
C6—C7—H7	119.8	O2—C18—H18B	109.5
C8—C7—H7	119.8	H18A—C18—H18B	109.5
O3—C8—C9	124.79 (15)	O2—C18—H18C	109.5
O3—C8—C7	114.39 (13)	H18A—C18—H18C	109.5
C9—C8—C7	120.82 (13)	H18B—C18—H18C	109.5
C8—C9—C10	119.77 (14)	O3—C19—H19A	109.5
C8—C9—H9	120.1	O3—C19—H19B	109.5
C10—C9—H9	120.1	H19A—C19—H19B	109.5
C9—C10—C1	121.47 (13)	O3—C19—H19C	109.5
C9—C10—C5	119.58 (12)	H19A—C19—H19C	109.5
C1—C10—C5	118.93 (12)	H19B—C19—H19C	109.5
O1—C11—C12	120.53 (13)		
			
C18—O2—C2—C1	5.6 (2)	C2—C1—C10—C5	0.6 (2)
C18—O2—C2—C3	−172.22 (13)	C4—C5—C10—C9	−179.51 (12)
C10—C1—C2—O2	−175.97 (12)	C6—C5—C10—C9	−0.7 (2)
C10—C1—C2—C3	1.7 (2)	C4—C5—C10—C1	−1.44 (19)
O2—C2—C3—C4	174.76 (12)	C6—C5—C10—C1	177.42 (13)
C1—C2—C3—C4	−3.1 (2)	C4—C3—C11—O1	−44.9 (2)
O2—C2—C3—C11	−2.86 (19)	C2—C3—C11—O1	132.68 (15)
C1—C2—C3—C11	179.26 (13)	C4—C3—C11—C12	133.39 (14)
C2—C3—C4—C5	2.3 (2)	C2—C3—C11—C12	−48.98 (19)
C11—C3—C4—C5	179.99 (13)	O1—C11—C12—C17	151.86 (15)
C3—C4—C5—C10	0.0 (2)	C3—C11—C12—C17	−26.5 (2)
C3—C4—C5—C6	−178.83 (14)	O1—C11—C12—C13	−26.7 (2)
C4—C5—C6—C7	179.15 (15)	C3—C11—C12—C13	154.93 (14)
C10—C5—C6—C7	0.3 (2)	C17—C12—C13—C14	2.1 (2)
C5—C6—C7—C8	−0.1 (3)	C11—C12—C13—C14	−179.24 (14)
C19—O3—C8—C9	−5.8 (2)	C12—C13—C14—C15	−0.8 (3)
C19—O3—C8—C7	173.98 (16)	C13—C14—C15—C16	−1.7 (2)
C6—C7—C8—O3	−179.71 (15)	C13—C14—C15—Cl1	178.11 (13)
C6—C7—C8—C9	0.1 (2)	C14—C15—C16—C17	2.8 (2)
O3—C8—C9—C10	179.38 (13)	Cl1—C15—C16—C17	−177.04 (12)
C7—C8—C9—C10	−0.4 (2)	C13—C12—C17—C16	−1.0 (2)
C8—C9—C10—C1	−177.32 (13)	C11—C12—C17—C16	−179.62 (14)
C8—C9—C10—C5	0.7 (2)	C15—C16—C17—C12	−1.4 (2)
C2—C1—C10—C9	178.63 (13)		

### Hydrogen-bond geometry (Å, °)

**Table:**

*D*—H···*A*	*D*—H	H···*A*	*D*···*A*	*D*—H···*A*
C18—H18C···O3^i^	0.96	2.51	3.460 (2)	171

Symmetry codes: (i) *x*−1, *y*, *z*−1.
